# Treatment Efficacy of Chuang Ling Ye, a Traditional Chinese Herbal Medicine Compound, on Idiopathic Granulomatous Mastitis: A Randomized Controlled Trial

**DOI:** 10.1155/2020/6964801

**Published:** 2020-06-29

**Authors:** Jing-xian Xue, Bei Ye, Shun Liu, Si-han Cao, Wei-he Bian, Chang Yao

**Affiliations:** Affiliated Hospital of Nanjing University of Chinese Medicine, Nanjing 210029, China

## Abstract

**Objective:**

To explore whether Chuang Ling Ye (CLY), a traditional Chinese herbal medicine compound, could improve the treatment of idiopathic granulomatous mastitis (IGM) via decreasing inflammatory response.

**Methods:**

Herein, 40 patients with IGM who had wounds requiring dressing change were enrolled and randomly divided into two groups: the CLY group and the control group. The size of the neoplasm and pain score of patients were followed-up for 4 weeks. Local tissues were taken during dressing change and examined by commercial enzyme-linked immunosorbent assay (ELISA) kits. The levels of inflammatory markers, including interleukin-1*β* (IL-1*β*), IL-2, IL-6, interferon gamma (IFN-*γ*), and tumor necrosis factor-*α* (TNF-*α*) were measured.

**Results:**

After treatment, the size of the neoplasm in the CLY group was significantly smaller than that in the control group (14.28 cm ± 8.96 cm vs. 21.14 cm ± 0.12 cm, *P*=0.038), and the pain scores were markedly reduced (*P*=0.004). Besides, CLY downregulated the expression levels of IL-1*β*, IFN-*γ*, and TNF-*α*.

**Conclusion:**

External use of CLY could reduce the neoplasm of IGM by inhibiting local inflammation. This trial is registered with ChiCTR1800017744.

## 1. Introduction

Idiopathic granulomatous mastitis (IGM) is a rare benign inflammatory breast disease with an estimated incidence of 2.4 per 100,000 women and 0.37% in the United States, while its incidence is higher among Asian populations [1]. The leading symptom of IGM is a painful neoplasm. Other symptoms are hyperemia, areolar, fistula, and ulceration [2]. IGM is diagnosed by histopathology only. The disease is characterized by formation of granuloma in combination with a localized infiltrate of multinucleated giant cells, lymphocytes, and plasma cells [3]. The etiopathogenesis of IGM reported in the literature included breast dysplasia, nipple deformity, smoking, and taking psychotropic drugs, leading to abnormal secretion of lipid substances in mammary duct endothelium, blockage of ducts caused by accumulation of secretions, and chronic inflammation [4], which suggested that inhibiting inflammation may be effective in the treatment of IGM.

In modern medicine, management of IGM cases remains controversial with proponents of initial surgical or medical therapies and each one has its associated complications, which may be worse than the original symptoms of IGM [5]. The main treatment option for IGM is surgery. In acute stage, antibiotics and surgical incision can be used, and the surgery can be performed after the neoplasm shrinks. If the neoplasm is small, local resection of the neoplasm can be carried out; if the neoplasm is large, a simple mastectomy can be conducted as well [6]. Versluijs-Ossewaarde et al. [7] found that 79% of patients were treated without removing the diseased catheter, while 28% were treated after removing the diseased catheter. Therefore, the main target of surgical treatment is to completely and thoroughly remove the lesions in the large lacteal duct avoiding recurrence. Lei et al. [8] demonstrated that surgical management have a high complete remission/resolution (CR) rate with a relatively low recurrence rate, with or without steroids. Thus, it is highly appropriate for patients requiring rapid remission. However, for patients with concerns about surgical scarring, oral steroids could be an acceptable option.

Traditional Chinese medicine (TCM) has significantly attracted scholars' attention for treating breast diseases [9]. For instance, Chuang Ling Ye (CLY), a traditional Chinese herbal medicine compound, is composed of rhubarb, safflower, *Abelmoschus manihot*, and *Terminalia* chebula. CLY has been used in clinical practice for promoting wound healing and reducing wound secretion since the 1990s. A previous study found that CLY has an appropriate anti-inflammatory effect, as well as an ability to promote angiogenesis [10–12]. We, in the present research, attempted to explore whether CLY could improve the treatment of IGM via reducing inflammatory response.

## 2. Patients and Methods

### 2.1. Design

This was a randomized controlled trial (RCT) and was conducted in accordance with the CONSORT-SPI 2018 checklist. Eligible patients were randomized 1 : 1 to either the CLY group or control group.

### 2.2. Study Subjects

#### 2.2.1. Inclusion Criteria

- Patients who were pathologically confirmed with IGM
- Patients who aged 18–60 years old
- No hormone or immunosuppressive therapy was used within one month before admission

#### 2.2.2. Exclusion Criteria

- Pregnant or lactating women
- Patients with allergic constitution or allergy to CLY
- Patients who would like to withdraw consent, or if they failed to adhere to the research protocol, the individual's participation was suspended, or withdrawal was recorded

### 2.3. Intervention

#### 2.3.1. CLY Group

After incision and drainage or self-ulceration of abscess, treatment was conducted for 4 weeks, including wound dressing with 30 ml CLY that flushed and drained with CLY gauze strip twice a week.

#### 2.3.2. Control Group

After incision and drainage or self-ulceration of abscess, treatment was carried out for 4 weeks, involving wound dressing with 30 ml saline that flushed and drained with saline gauze strip twice a week.

#### 2.3.3. Preparation of CLY

CLY was herein composed of 200 g of rhubarb, 100 g of safflower, 50 g of *Abelmoschus manihot*, and 100 g of *Terminalia* chebula (Table 1). Among the abovementioned four flavors, *Abelmoschus manihot* was heated and refluxed twice with 75% ethanol for 1.5 h each time. For the other three flavors, water was added and decoction was done twice. After that, the filtrate was combined, it was concentrated to the extract with a relative density of 1.15 (80°C), and ethanol was added to make the alcohol content 70%. Then, it was allowed to settle, the supernatant was taken to recover ethanol, and it was concentrated to about 900 mL. The *Abelmoschus manihot* was added; while it is hot, it was mixed well and settled for 24 h, and water was added to 1000 mL [12].

#### 2.3.4. Quality Control

Analysis of components of CLY was carried out by ultraperformance liquid chromatography-tandem mass spectrometry (UPLC-MS/MS). Accurately, 1 mL of CLY was measured, it was concentrated to dryness under vacuum, and it was dissolved into 10 mL to obtain the test solution. After shaking the test solution, it was centrifuged at 16,000 rpm for 10 min, it was filtered with a 0.22 *μ*M pore size filter, 190 *μ*L of continuous filtrate was taken, and 10 *μ*L of caffeic acid was added. The mobile phase consisted of acetonitrile and 0.2% ammonium acetate. The gradient program was produced by increasing the rate of acetonitrile from 25% to 80% and decreasing the rate of ammonium acetate from 75% to 20% for 13 min. Mass spectrometry analyses were performed on a UHPLC system (Acquity H-Class, Waters Corp., Milford, MA, USA) with a binary solvent manager, a column manager, and a sample manager. The sample was separated on a Waters Acquity HSS T3 column (100 mm × 2.1 mm, 1.8 *μ*m, Waters Corp.), and the column temperature was set at 40°C. The mobile phase consisted of acetonitrile (A) and water containing 0.05% formic acid (B) with a flow rate of 0.25 mL/min. For UHPLC-MS/MS analysis, a Waters QqQ-MS (Xevo TQ-D, Waters Corp.) was connected to the Waters UHPLC instrument via an electrospray ionization (ESI) source. Analytes were quantified by multiple reaction monitoring (MRM) in negative ionization mode with argon as the collision gas.

### 2.4. Outcome Measures

#### 2.4.1. Primary Outcome Measures

Neoplasm size was measured by the area of the neoplasm. The area of the neoplasm was photographed at the time of treatment. Finally, the ImageJ 16.0 software was used to measure the area of the neoplasm.

#### 2.4.2. Pain Score

The Visual Analog Scale pain scoring-system was employed, in which 10-point indicated the most painful condition, while 0-point denoted no painful condition.

#### 2.4.3. Inflammatory Factors

At days 1, 15, and 29, the levels of inflammatory factors, such as interleukin-1*β* (IL-1*β*), IL-2, IL-6, interferon gamma (IFN-*γ*), and tumor necrosis factor-*α* (TNF-*α*) were measured using commercial enzyme-linked immunosorbent assay (ELISA) kits (Hangzhou Multisciences Biotech Co, Ltd., Hangzhou, China).

### 2.5. Sample Size

We collected baseline data of 10 IGM patients who were treated in our hospital with CLY and were recovered after 3 mouths, in addition to data extracted from previous studies (Chirapapha et al., 2018, [2]). To account for a potential drop-out rate of up to 20% at 28 days, we planned to randomize 45 patients.

### 2.6. Randomization

Patients were randomly assigned to study groups by nonstratified randomization. The random sequence was generated using the random number function of Microsoft® Excel software. The randomization list was kept on a password-secured computer. All patients, study site personnel, raters, and contract research organization staff were blinded to group assignment.

### 2.7. Research Ethics and Informed Consent

The research protocol was approved by the Institutional Review Board of the Affiliated Hospital of Nanjing University of Traditional Chinese Medicine (Nanjing, China). From September 2018 to October 2019, patients who were admitted from the Breast Disease Department of our hospital were screened and enrolled in the current study, and all the eligible patients signed the written informed consent form prior to commencing the study. The research protocol was registered at the Chinese Clinical Trial Registry (reg. no. ChiCTR1800017744).

### 2.8. Statistical Analysis

Continuous data were compared using Student's t-test. Groups were compared using the t-test or Student's t-test, as appropriate based on the data distribution. Categorical variables were expressed as percentages. Groups were compared using the chi-square or Fisher's exact test, as appropriate based on the expected counts. All statistical analyses were performed using SPSS 17.0 software (IBM, Armonk, NY, USA).

## 3. Results

### 3.1. Analysis of the Main Medicinal Components of CLY

**Figure 1:**
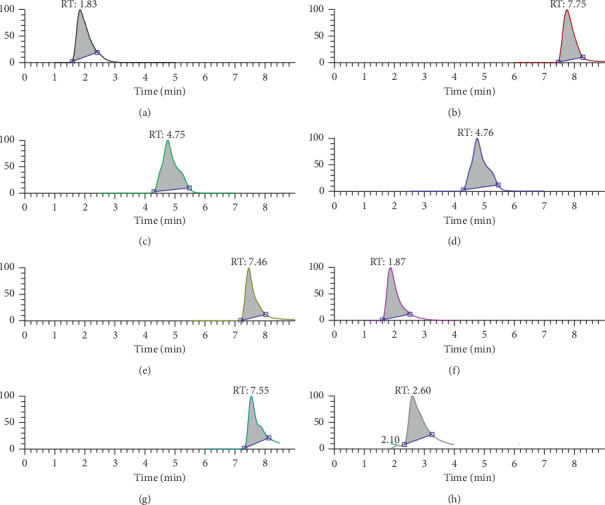
Analysis of the main components of CLY. (a) Caffeic acid, (b) emodin, (c) rhein, (d) physcion, (e) aloe-emodin, (f) hydroxysafflor yellow A, (g) kaempferide, and (h) hyperoside.

**Figure 2:**
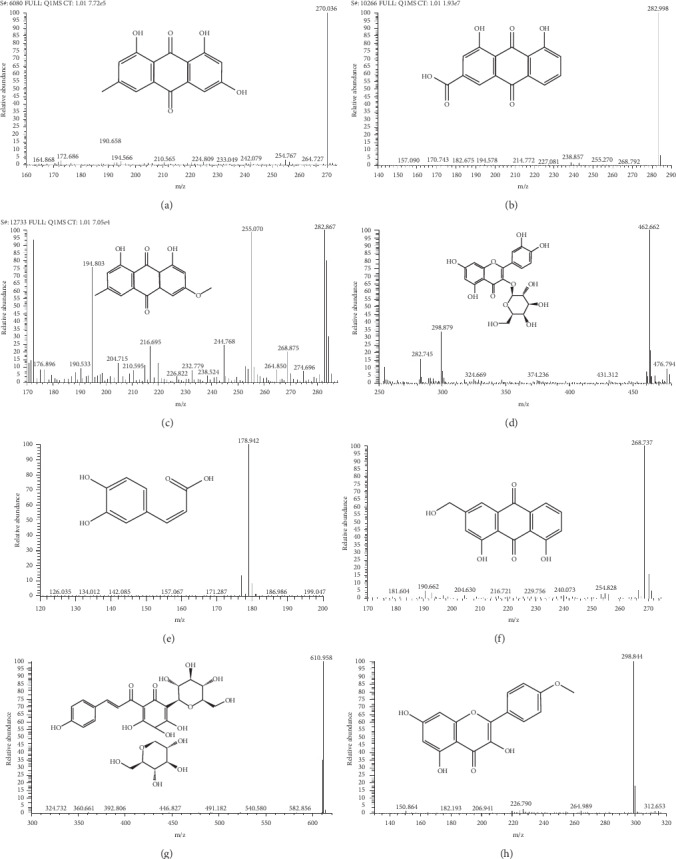
Chromatogram. (a) emodin, (b) rhein, (c) physcion, (d) hyperoside, (e) caffeic acid, (f) aloe-emodin, (g) hydroxysafflor yellow A, and (h) kaempferide.

The main components of CLY were analyzed by UPLC-MS/MS. CLY contained emodin (0.09178 mg/ml), rhein (0.25078 mg/ml), physcion (0.15954 mg/ml), aloe-emodin (0.09472 mg/ml), hydroxysafflor yellow A (0.61545 mg/ml), kaempferide (0.001943 mg/ml), and hyperoside (0.92247 mg/ml) (Figures 1 and 2).

### 3.2. Patients' Baseline Characteristics

**Figure 3:**
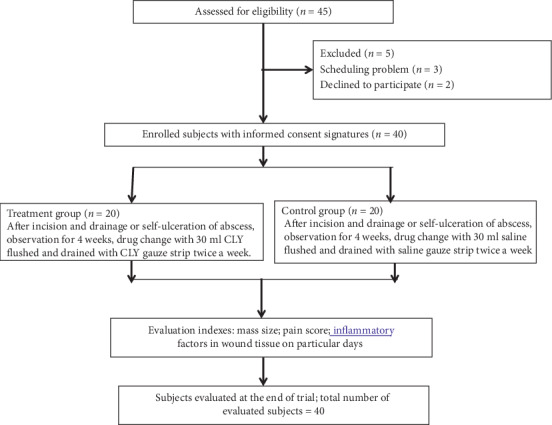
CONSORT followchart of the clinical study.

From September 2018 to October 2019, 45 patients were screened for eligibility and 5 of whom were ineligible (Figure 3). Of the remaining 40 patients, 20 were assigned to the CLY group and 20 to the control group. In the CLY group, 4 patients received wound healing at day 29, while 2 patients in the control group received wound healing at day 29. The patients' age ranged from 26 to 50 years old, and they all were female. There was no significant difference in the patients' age, weight, and breastfeeding month between the two groups. During the treatment period (within 4 weeks), no oral hormones or antibiotics were given to the two groups (Table 2).

### 3.3. Size of Neoplasm

There were neoplasms in the two groups before treatment. At day 1, the size of the neoplasm in the control group was 34.2–48.3 cm^2^, and that was 34.0–47.4 cm^2^ in the CLY group. There was no significant difference in the size of the neoplasm between the two groups (*P*=0.865). At day 15, both groups had a smaller neoplasm than before. At day 29, the size of the neoplasm in the CLY group was remarkably smaller than that in the control group (*P*=0.001 < 0.05). This suggested that IGM was a self-healing disease. In the presence of wound (natural rupture or incision and drainage), regular dressing change and timely drainage could reduce the size of the neoplasm. The use of CLY during dressing change could markedly decrease the size of the neoplasm (14.28 + 8.96 cm^2^ vs. 21.14 + 10.12 cm^2^, *P*=0.038) (Table 3).

### 3.4. Pain Score

In the current study, we found that the patients' mean pain score was 6-point before treatment. Pain affected patients' daily life. There was no significant difference in pain score between the two groups at day 1 (*P*=0.829). After treatment, the pain score significantly decreased, which was consistent with the trend of decreasing the size of the neoplasm. After treatment, the pain score in the CLY group was noticeably lower than that in the control group (*P*=0.004 < 0.05), highlighting that the use of CLY had a relieving effect on the patients' pain (Table 4).

### 3.5. Inflammatory Factors

The findings of the present research revealed that there was no significant difference in the levels of IL-1*β*, IFN-*γ*, and TNF-*α* between the two groups at day 1, while a significant difference in the levels of these factors was noted between the control group and the CLY group after treatment. It was unveiled that CLY could reduce the neoplasm by inhibiting inflammation. IL-6 also plays a significant role in inflammatory response [11, 24]; however, there was no significant difference between the two groups after treatment in the present experiment (*P*=0.978 > 0.05). The outcomes of the current research uncovered that there was no significant difference in IL-2 level between the two groups before treatment. After treatment, the *P* value of the difference related to IL-2 level between the control group and the CLY group was 0.057, and there were still some differences although that was greater than 0.05 (Table 5). Since CLY was composed of *Abelmoschus manihot*, it may play a role in immune response to IGM.

During treatment, there were patients who received wound healing in the two groups; thus, no samples were taken from these patients. There were 2 and 4 cases who received wound healing in the control group and the CLY group, respectively.

## 4. Discussion

IGM can mimic two very frequent breast disorders, breast carcinoma, and breast abscess. To date, a limited number of RCTs were reported, and the majority of publications were retrospective studies and case series [25, 26]. A retrospective cohort study showed that there was no significant difference among treatment modalities in terms of time-to-healing and recurrence of disease (Chirapapha et al. 2018). The current RCT presented a new effective therapy for IGM. The results of the present research revealed that CLY could promote the shrinkage and healing of plasma cell mastitis and decrease the pain in the course of disease. Measurement of the levels of inflammatory factors unveiled that CLY could inhibit the levels of IL-1*β*, IFN-*γ*, and TNF-*α*.

For the neoplasm treatment, there are several therapeutic strategies as follows, in which surgical treatment is the main method for a simple neoplasm (Chirapapha et al. 2018). However, several patients have a neoplasm with abscesses or fistulas. At this time, the main idea is to reduce the size of the neoplasm and heal the wound, and then removing the lesions of the breast tissue should be followed. For the reduction of size of tumors, the mainstream method of Western medicine is hormone therapy [27]. In a meta-analysis [8], 106 patients were analyzed for surgical managements, oral steroids, and oral steroids + surgical managements, respectively. The pooled estimates for their CR rate were 90.6%, 71.8%, and 94.5%. The pooled estimates for recurrence rate were 6.8%, 20.9%, and 4.0%, respectively. The present study uncovered that IGM had a tendency of self-healing, which was consistent with Lei et al.'s findings [8]. However, we also found that the severity of pain should be take into consideration in treatment of IGM. Our results indicated that CLY had a significant effect on pain relief in IGM patients.

The use of herbal TCM to treat various diseases has an interesting philosophical background with a long history, while it received increasing skepticism due to the lack of evidence-based efficiency as shown by high quality trials. Our previous study revealed that CLY had an acceptable anti-inflammatory effect, promoting blood circulation and wound healing [13]. In the present study, we found that CLY could inhibit the levels of inflammatory factors, such as TNF-*α*, IL-1*β*, and IFN-*γ*, suggesting that CLY could promote wound healing by inhibiting the levels of inflammatory factors.

IL-2 is a pleiotropic cytokine produced after antigen activation that plays pivotal roles in the immune response [28]. IL-2 can promote the generation of T effector cells including those of the Th1 and Th2 lineages, while inhibiting the differentiation of Th17 cells [29]. Although there is no literature supporting the role of IL-2 in granulomatous mastitis, our experiment found that there was no significant difference between the two groups before treatment. After treatment, the *P* value of IL-2 was 0.057, and there were still some differences although that was greater than 0.05. This indicated that CLY may play a substantial role in the immune response to IGM, while further study needs to be conducted to confirm our findings.

There are a number of limitations in the current study. First, lack of long-term follow-up of recurrence rate was tangible. Secondly, IL-6 is also an important cytokine, and it was previously reported that IL-6 plays a key role in the development of IGM [13, 30]. Thirdly, our findings unveiled that CLY had insignificant influence on IL-6 level, which is worthy of further discussion.

In summary, our findings proved that CLY could promote the healing of the neoplasm and alleviate the pain in patients with IGM.

## Figures and Tables

**Table 1:** CLY composition.

Name (botanical, common Pinyin names), traditional daily dose (grams)	Active compounds	Clinical and pharmacological effects	Adverse effects/toxicity
*Abelmoschus manihot* (L.) Medik, Huang Shukuihua, 10–30 g	The known chemical component of *Abelmoschus manihot* is mainly hyperoside [13]	*Abelmoschus manihot* treats some kidney diseases. It protects renal tubular cells via inhibition of ROS-ERK1/2-NLRP3 pathway [13–15]	No reported adverse events
*Rheum palmatum* L., rhubarb root and rhizome, Da Huang, 3–15 g	The rhubarb root contains aloe-emodin, rhein, emodin, chrysophanol et al. [16]	It has been used for a variety of conditions, such as constipation, chronic renal failure, and upper gastrointestinal bleeding. It has antimicrobial and antioxidative effects [17, 18]	The toxicity includes hepatotoxicity and nephrotoxicity. No adverse reactions were observed in mice with a dose of 400 mg/kg [19]
*Carthamus tinctorius* L., safflower, Hong Hua, 3–10 g	The flowers of safflower contain kaempferol and hydroxysafflor yellow [20]	It reduces immune inflammatory response [14]. *Carthamus tinctorius* (safflower) oil is used as a dietary supplement for weight loss and antioxidant [21]	3 patients had a diagnosis of acute liver failure, while consumed safflower as a dietary supplement for weight loss [21]
*Terminalia chebula* Retz., Ke Zi, 3–10 g	Contains chebulin and hyperoside [16, 22]	It is used in asthma, ulcers, gout, heart, and bladder diseases [23]	No reported adverse events

**Table 2:** Baseline characteristics of the participants.

	Control group	CLY group	*P* value
Age (years)	34.65 ± 7.49	34.35 ± 4.42	0.878
Height (cm)	158.6 ± 4.58	159 ± 3.6	0.760
Weight (kg)	57.7 ± 6.81	57.3 ± 6.59	0.851
BMI (kg/m^2^)	22.5 ± 2.69	21.78 ± 2.36	0.374
Lactation (month)	9.55 ± 5.08	8.95 ± 4.55	0.696

**Table 3:** Comparing the size of the neoplasm (in cm^2^) between the two groups.

	Control group	CLY group	Estimated group difference (95% confidence interval)	*P* ^a^
Day 1	41.09 ± 4.00	41.33 ± 4.66		0.865
Day 15	29.14 ± 7.71	27.46 ± 9.25	−1.92 (−7.61, 3.77)	0.489
Day 29	21.14 ± 10.12	14.28 ± 8.96	−7.1 (−13.75, −0.45）	0.038
*P* ^b^	0.001	0.001		

^a^Comparing the two groups. ^b^Comparing at day 29 with that at day 1.

**Table 4:** Comparison of the pain score between the two groups.

	Control group	CLY group	Estimated group difference (95% confidence interval)	*P* value
Day 1	5.45 ± 1.00	5.90 ± 0.72		0.849
Day 15	3.85 ± 1.18	3.50 ± 1.05	−0.5 (−1.25, 0.25)	0.180
Day 29	2.20 ± 1.20	1.15 ± 0.88	−1.10 (−1.79, −0.41)	0.004

**Table 5:** Comparing the levels of inflammatory factors between the two groups.

Inflammatory factor (pg/ml)		Control group	CLY group	Estimated group difference (95% confidence interval)	*P*
TNF-*α*	Day 1	248.34 ± 53.56	243.8 ± 78.15		0.831
	Day 15	264.19 ± 95.67	159.48 ± 72.25	−85.47 (−149.65, −21.28)	0.012
	Day 29	200.20 ± 76.81	110.95 ± 56.91	−81.59 (−156.45, −6.73)	0.035

IFN-*γ*	Day 1	36.95 ± 15.42	50.41 ± 37.06		0.145
	Day 15	36.81 ± 15.64	32.00 ± 27.46	−18.27 (−41.20, 4.65)	0.112
	Day 29	38.8 ± 23.53	16.35 ± 11.1	−45.78 (−68.58, −22.97)	0.001

IL-1*β*	Day 1	767.11 ± 235.88	763.43 ± 176.21		0.956
	Day 15	705.1 ± 205.51	370.56 ± 177.94	−0.50 (−1.25, 0.25)	0.180
	Day 29	723.8 ± 198.57	248.47 ± 119.62	−1.10 (−1.79, -0.41)	0.004

IL-2	Day 1	224.91 ± 97.41	234.56 ± 93.62		0.751
	Day 15	167.79 ± 124.42	111.96 ± 87.89	−65.48 (−159.64, 28.69)	0.162
	Day 29	114.05 ± 64.86	48.47 ± 27.53	−89.27 (−181.55, 3.02)	0.057

IL-6	Day 1	144.42 ± 47.67	132.38 ± 60.68		0.489
	Day 15	88.06 ± 39.60	66.34 ± 38.2	−9.67 (−50.02, 30.67)	0.622
	Day 29	50.64 ± 20.12	36.91 ± 16.54	−0.65 (−49.53, 48.23)	0.978

## Data Availability

The data used to support the findings of this study are available from the corresponding author upon request.

## Abbreviations

CLY: Chuang Ling Ye
IL-2: Interleukin-2
IL-6: Interleukin-6
IL-1*β:*: Interleukin-1*β*
IFN-*γ*: Interferon gamma
TNF-*α*: Tumor necrosis factor-*α*
IGM: Idiopathic granulomatous mastitis.
